# Non-toxic Megacolon Secondary to High-Grade Large-Bowel Obstruction

**DOI:** 10.7759/cureus.21580

**Published:** 2022-01-25

**Authors:** Nathan D Mullen, Hunter Thurn, Eric Karr, Kathryn M Burtson

**Affiliations:** 1 Internal Medicine, Wright-Patterson Medical Center/Wright State University, Dayton, USA

**Keywords:** abdominal distention, large bowel perforation, bowel ischemia, bowel obstruction, megacolon

## Abstract

A 92-year-old male presented from an outside hospital for treatment of a chronic obstructive pulmonary disease exacerbation (COPD) and subsequently developed worsening abdominal distention with pain during the course of his hospitalization. He was found to have a high-grade large-bowel obstruction with a dilated colon of 20 cm measuring upward. The patient ultimately underwent a hemicolectomy to prevent bowel ischemia and reformation of another volvulus. We present this case to elucidate the need for vigilant monitoring in patients with chronic bowel obstruction due to lack of typical symptoms, to demonstrate a successful management approach, and to exhibit an extreme example of the resulting megacolon.

## Introduction

Volvuli are the most common benign etiology of large-bowel obstructions and account for 1%-7% of all cases [1,2]. Typical presenting signs and symptoms include abdominal pain, obstipation, and nausea with or without vomiting along with abdominal distention and abdominal tenderness to palpation. The frequency of these symptoms varies; however, a review of 300 patients in one study had reported abdominal pain and nausea in 92% and 82% of patients, respectively [3]. Computed tomography (CT) is the preferred diagnostic imaging study due to its high sensitivity and specificity in detecting large-bowel obstructions. Using diagnostic image studies, bowel obstructions can be classified as either high-grade indicating no passage of fluid or gas beyond an obstruction point or partial indicating some passage of fluid or air [4]. The development of non-toxic megacolon can be a complication of large-bowel obstructions and, if left untreated, carries high rates of morbidity and mortality [5,6]. In this study, we present a case of an elderly man with the development of significant non-toxic megacolon secondary to a high-grade large-bowel obstruction.

## Case presentation

A 92-year-old male with a medical history significant for COPD, atrial fibrillation, and previous remote partial left hemicolectomy with anastomosis presented as a transfer for continued treatment of COPD exacerbation. Vitals at the time of initial presentation were within normal limits, and the patient's oxygen saturation was greater than 92% on room air. Physical exam was notable for wheezing along with abdominal distention but without tenderness to palpation. He was treated with glucocorticoids and nebulized ipratropium bromide/albuterol for his COPD exacerbation.

Two days into his admission, the patient developed abdominal pain with reduced stool output and minimal passage of gas. Upon further assessment, the patient stated his abdominal distention was typically normal and reported regular bowel movements at home without blood. He also reported multiple previous surgeries "to get rid of the gas" that suggested he was having chronic issues with bowel obstruction; however, the treatment team was unable to obtain these medical records. During the time of the patient's initial onset of abdominal pain, vitals were within normal limits. Physical exam was notable for severe and interval progression of abdominal distention but without abdominal tenderness or peritoneal signs. Lactic acid was elevated at 3.5 mmol/L (normal range: 0.5-2.2 mmol/L).

**Figure 1:**
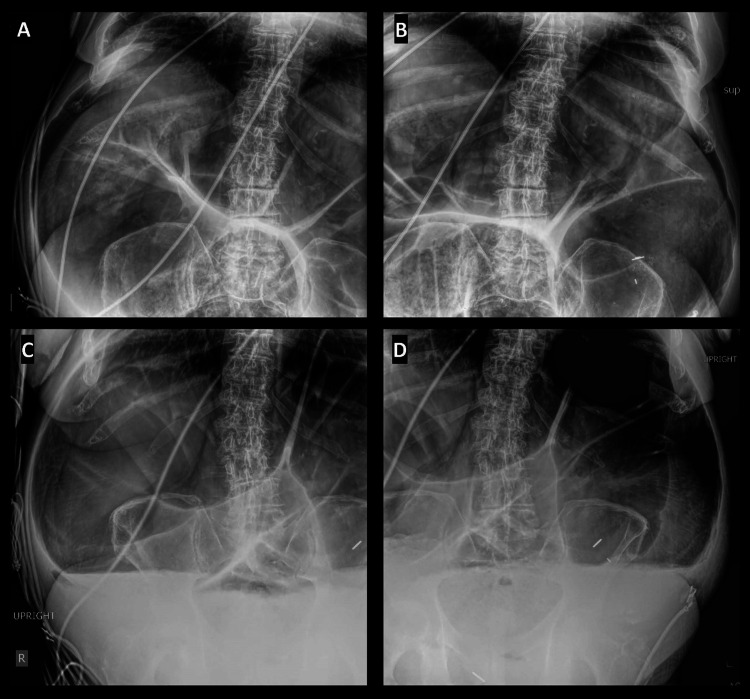
Abdominal series x-ray demonstrating extensively distended bowel (A) Right supine view. (B) Left supine view. (C) Right upright view. (D) Left upright view.

**Figure 2:**
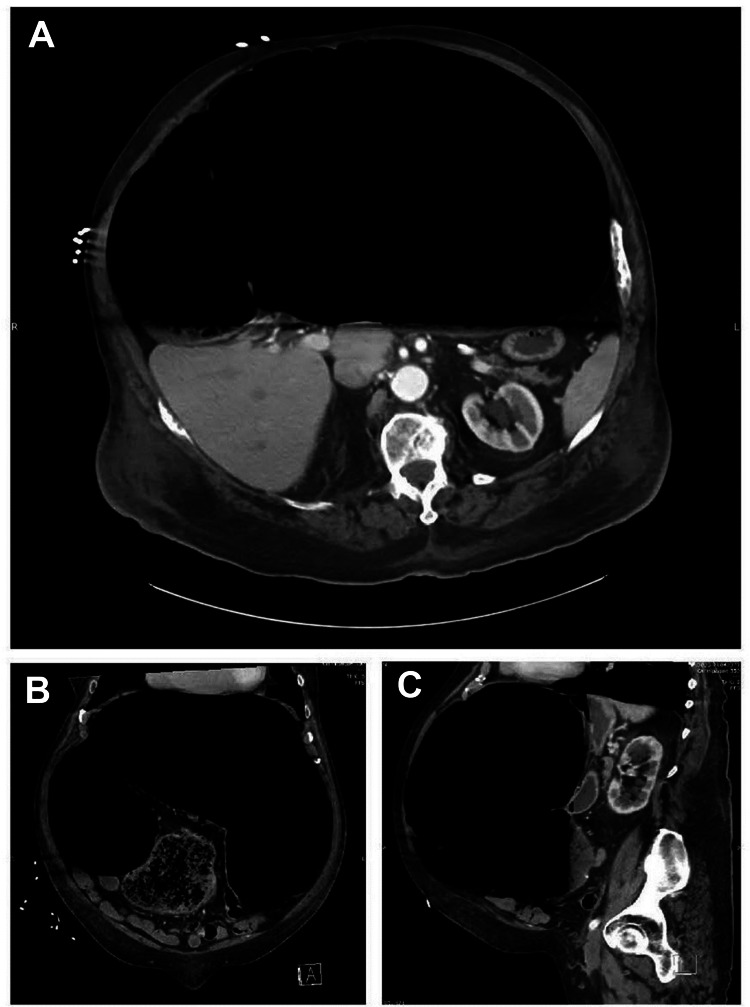
Non-contrast computed tomography of the abdomen and pelvis demonstrating massively dilated large bowel measuring up to 20 cm (A) Transverse view. (B) Coronal view. (C) Sagittal view.

**Figure 3:**
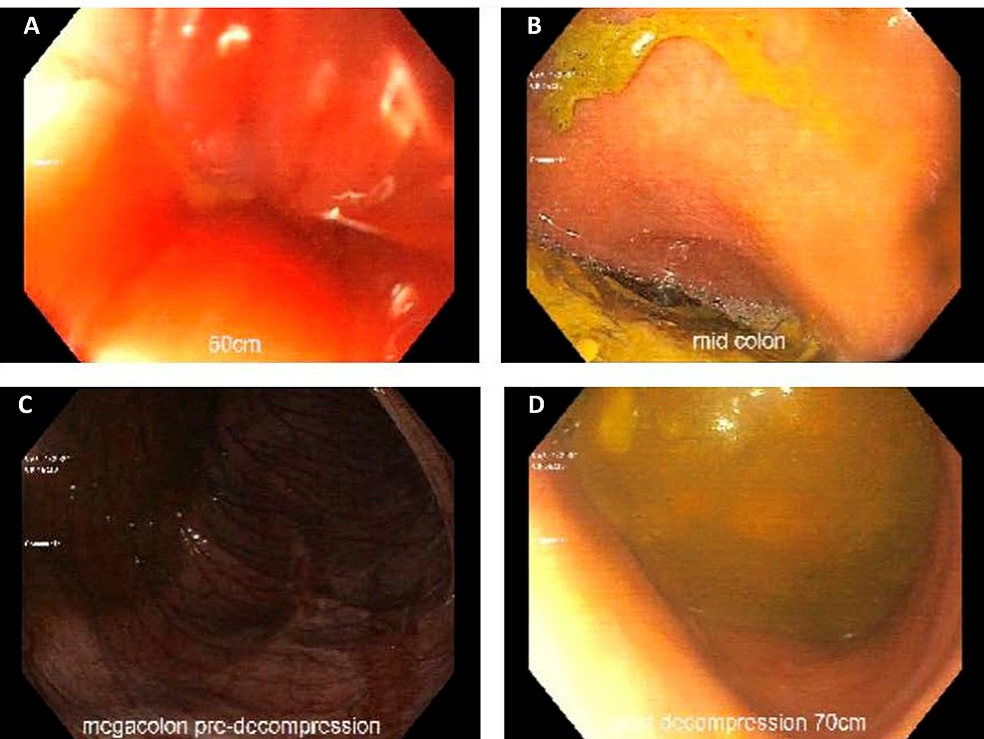
Endoscopic pictures of colon demonstrating dusky, purplish color pre-decompression with the return of normal-appearing mucosa post-decompression (A) Distal colon. (B) Mid-colon. (C) Dusky appearing megacolon pre-decompression. (D) Normal-appearing mucosa post-decompression.

An abdominal series x-ray demonstrated extensively distended bowel suggestive of large-bowel obstruction (Figure 1). Non-contrast CT of the abdomen demonstrated a massively dilated large bowel measuring up to 20 cm with high-grade colonic obstruction (Figure 2). A nasogastric and rectal tube were immediately placed, and the patient was switched to nil per os (NPO). Surgery and gastroenterology were consulted who coordinated an emergent sigmoidoscopy in the setting of high perforation risk, with decompression of the colon. During sigmoidoscopy, the proximal colon appeared to have a dusky, purplish appearance with marked improvement to pinkish mucosa and a normal-appearing mucosal pattern after decompression with air suction (Figure 3). Despite colonic decompression and conservative measures, the patient experienced interval worsening of abdominal distention without stool output, and he was taken to the operating room for a total abdominal colectomy with end ileostomy. After a successful procedure, the patient recovered and was discharged to a rehabilitation center.

## Discussion

A diagnosis of megacolon is made when the colon diameter is found to measure >12 cm and is classified as either acute or chronic. Acute megacolon in the setting of inflammation is known as toxic megacolon, whereas acute megacolon without known colonic disease is known as Ogilvie’s syndrome [7]. Chronic megacolon is typically caused by bowel dysfunction from neurological or muscular disorders. We hypothesize that our patient likely had significant bowel dysfunction from his previous hemicolectomy with anastomosis, resulting in an insidious chronic bowel dysfunction with obstruction and development of non-toxic megacolon.

For patients with acute abdominal pain with distention, x-ray imaging of the abdomen should be obtained to assess for an obstructive process along with follow-up CT imaging to determine the location of the obstruction. Obtaining a lactic acid level can be useful in determining whether bowel ischemia is occurring. Our patient’s lactic acid was elevated, with CT imaging remarkably impressive for dilated large bowel measuring 20 cm, with what appeared to be compression of organs against the posterior wall concerning for abdominal compartment syndrome.

There are limited published case reports on patients with chronic non-toxic megacolon, making morbidity and mortality rates difficult to discern. Multiple studies have suggested an increase in perforation rates with the dilated colon as little as 9 cm, with a greater significant risk of perforation with cecal diameters greater than 10-12 cm [8,9]. One particular study suggested a 23% perforation rate in patients with non-mechanical cecal dilation greater than 14 cm [10]. The mortality rate has been estimated to be 20%-50% once perforation occurs [11,12]. Other complications are associated with megacolon and include peritonitis with the development of sepsis, mesenteric ischemia, and compression of the vena cava [13-15].

The development of megacolon, whether acute or chronic, should be quickly investigated and managed. Management of stable patients with the development of chronic megacolon is typically conservative and is directed at preventing ischemia and perforation. Pharmacologic approaches are often effective such as intravenous neostigmine, with alternative methods including colonic decompression [16]. If conservative measures fail, surgical intervention is indicated.

Our patient failed initial conservative measures, and he ultimately decided to pursue surgical intervention consisting of total colectomy with end ileostomy. Given the limited published studies on geriatric patients with the development of chronic non-toxic megacolon, this case report aims to demonstrate the clinical presentation and management strategies for this uncommon disease process.

## Conclusions

In conclusion, a diagnosis of megacolon should prompt thorough investigation with hastened management. While our patient was relatively stable and without signs of perforation, his markedly dilated bowel after failing conservative measures was significant enough for a total colectomy with end ileostomy to prevent an unfavorable outcome.
